# Melanoma – goes where it wants and does what it pleases. A case report of primary gastric melanoma

**DOI:** 10.1093/jscr/rjae246

**Published:** 2024-04-24

**Authors:** Matthew Corbitt, Vipul Vyas, Christian J Beardsley

**Affiliations:** School of Medicine & Dentistry, Griffith University, 1 Parklands Drive, Gold Coast, QLD 4222, Australia; Department of Surgery, Cairns & Hinterland Hospital and Health Service, 165 Esplanade, Cairns, QLD 4870, Australia; Department of Anatomical Pathology, Royal Brisbane and Women’s Hospital, Butterfield Street, Brisbane, QLD 4006, Australia; Department of Surgery, Cairns & Hinterland Hospital and Health Service, 165 Esplanade, Cairns, QLD 4870, Australia

**Keywords:** gastric, melanoma

## Abstract

Melanoma is a common global cancer, however, extracutaneous forms are most often from metastasis. Primary extracutaneous forms are rare, with primary gastric melanoma exceedingly so, with approximately only 50 cases reported worldwide in the literature. The mainstay of management is surgical resection with minimal data on adjuvant therapy. Its prognosis remains poor due to its aggressive behaviour and late presentation. Our case demonstrates a primary gastric melanoma presenting with gastric perforation requiring emergency operative management.

## Introduction

While cutaneous melanoma is a common malignancy globally, extracutaneous primary melanomas are not, with primary gastric melanomas being exceedingly rare. Our case demonstrates a primary gastric melanoma presenting as gastric perforation who underwent emergency operative management with good early outcome. It is prudent to be aware of other rare causes of upper gastrointestinal pathology when there are atypical findings.

## Case report

A 49-year-old male presented acutely to the emergency department after experiencing sudden escalation of left upper quadrant, epigastric and chest pain three hours prior. This was preceded by a 48-hour history of low-grade epigastric pain. He also describes 2–3 episodes of melaena two weeks prior. No nausea, vomiting, haematemesis or frank per-rectal bleeding. No history of infective or constitutional symptoms. Intermittent NSAID use over previous years, however, denies any in the past three weeks. No corticosteroid use. No other significant medical history, no allergies, ex-smoker and drinks alcohol socially.

**Figure 1:**
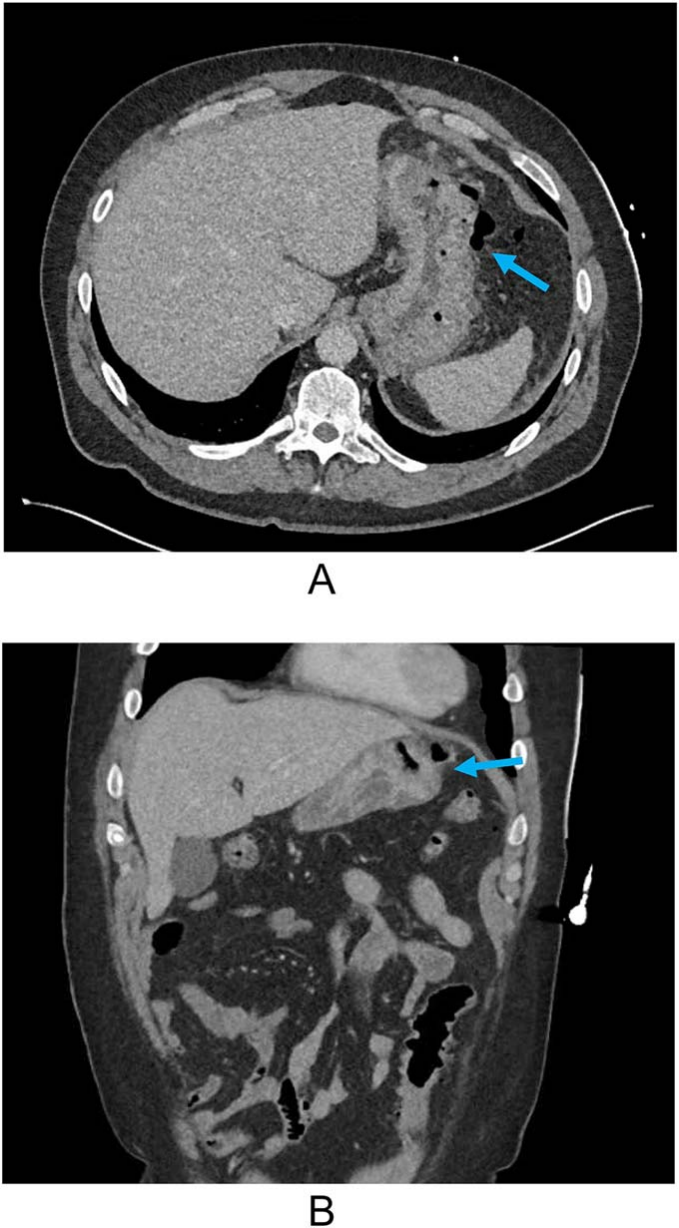
(A) Axial CT and (B) coronal reconstruction demonstrating perforated gastric ulcer at the greater region of the gastric body (arrows).

He was hypertensive on arrival (176/115 mmHg) but had otherwise normal vital signs. Abdominal examination yielded focal peritonism in the epigastrium and left upper quadrant and erect chest x-ray demonstrated free air. His initial blood results were only significant for a mild compensated metabolic acidosis and mildly elevated white cell count (11.9×10^9^/L, RR 4–11). His subsequent CT showed free intra-peritoneal air predominately in the upper abdomen with a penetrating ulcer on the greater region of the gastric body (Fig. 1A and B).

He was booked urgently for theatre and underwent exploratory laparotomy. Intra-operative findings included a large, penetrating and perforated gastric ulcer in the mid-body of the stomach. However, it was also surrounding by a large, fixed mass and fibrosis, highly suspicious for malignancy, which was confirmed by on-table gastroscopy. Exploration for potential intra-abdominal metastatic disease was negative and because of concern for malignancy and poor tissue quality, a subtotal gastrectomy with Roux-en-Y reconstruction was performed. The resection was non-oncological resection due to perforation.

The patient had an uncomplicated post-operative course and was discharged on day six with a proton-pump inhibitor and a course of oral antibiotics after receiving intravenous antibiotics and anti-fungal therapy during his admission. However, he was readmitted day eight post-operatively for six days with a left lower zone pneumonia and a reactive left sided pleural effusion that settled with conservative management.

**Figure 2:**
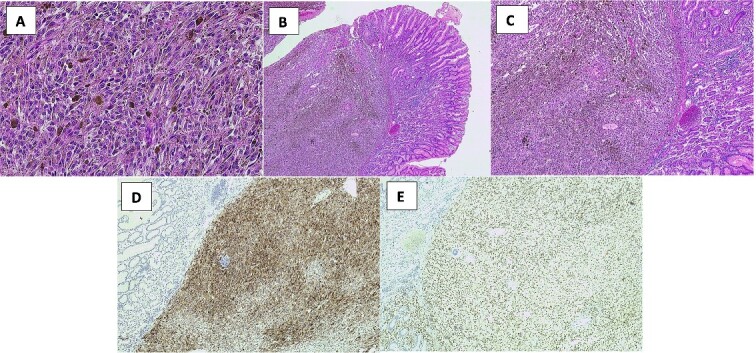
H&E and IHC stains of the histopathology specimens demonstrating malignant melanoma in the surgically resected specimen. (A) Malignant melanoma with cells showing atypical nuclei, prominent nucleoli and melanin pigment (high power H&E). (B) Normal small bowel mucosa with submucosal melanoma (low power H&E). (C) Submucosal melanoma with melanin pigment. (D) Melanoma cells positive for Melan-A. (E) Melanoma cells positive for SOX10.

Histopathology unexpectedly demonstrated malignant melanoma, 0/5 lymph nodes (Fig. 2). His molecular studies detected a BRAF V600E (Val600Glu) variant. No Helicobacter-like organisms were seen. The patient has no personal history of melanoma, had a negative skin check as an inpatient and had no family history of malignancy. Completion staging including FDG-PET did not demonstrate any metastatic disease.

The patient was discussed at an oncological multidisciplinary team meeting, was enrolled in a clinical trial for management of his disease and has recovered well post-operatively.

## Discussion

Melanoma is a common malignancy, with cutaneous melanoma incidence being 1.6% globally, and representing over 95% of all melanoma cases [1]. Metastasis is most commonly skin, subcutaneous tissue, bone, brain liver and lungs, and rarely gastrointestinal [1]. Even rarer is gastric metastasis. Primary gastric melanoma is exceedingly rare, accounting for only 1% of all extracutaneous melanomas [1]. The presentation is vague, and the diagnosis is often incidental, made via histological and immunohistopathological evidence, and exclusion of another source [1–3]. There are also no definite guidelines on the management of primary gastric melanoma due to the paucity of cases [1, 2].

A systematic review published in 2020 found that the average age of diagnosis was 63.4 years with a male predominance. The tumour was most frequently found in the gastric body and all those that were resected with partial gastrectomy showed improved survival. It displayed aggressive behaviour with median recurrence of 5 months, and only 12% of patients reached five-year survival. Another systematic review in 2020 demonstrated similar findings and suggested that it was not related to BRAF mutations [2].

The gold-standard treatment is still surgical resection and research has yet to demonstrate any significant overall survival benefit with adjuvant therapies, with treatment protocols being extrapolated from cutaneous melanoma research [1, 2]. However, those with detected BRAF variants represent a potential immunotherapy target.
